# Unveiling the prognostic role of FABP4 in early-onset colorectal cancer through big data analysis and preliminary clinical validation

**DOI:** 10.3389/fonc.2025.1689952

**Published:** 2026-01-13

**Authors:** Yu Wu, Weiwei Zou, Shengjun Zhang, Lipeng Zhao, Shaohua He, Fan Yao, Peilin Qing, Yixin Li, Jie Li, Xiao-Liang Xing

**Affiliations:** 1 Department of Clinical Laboratory, Hunan University of Medicine General Hospital, Hunan University of Medicine, Huaihua, Hunan, China; 2 School of Public Health and Emergency Management, School of Medical Laboratory Science, Hunan University of Medicine, Huaihua, Hunan, China; 3 Gynecological Oncology Department, Huaihua Central Hospital, Huaihua, Hunan, China; 4 Gastroenterology Department, Huaihua Central Hospital, Huaihua, Hunan, China

**Keywords:** FABP4, biomarker, risk model, therapeutic, EOCRC

## Abstract

**Background:**

Early-onset colorectal cancer (EOCRC), characterized by greater aggressiveness and advanced stage at diagnosis, is increasing globally. This study aimed to identify suitable prognostic biomarkers for EOCRC.

**Methods:**

Gene expression and clinical data from TCGA and GEO (GSE39582, GSE17536, and GSE17537) datasets were analyzed. Differential expression, univariate and multivariate Cox regression, and correlation analyses were performed. Immune status was evaluated using ESTIMATE and CONSENSUS TME algorithm. Immunotherapy and chemotherapy response was predicted via the TIDE and oncoPredict algorithm, respectively. Candidate signatures were validated in clinical samples from three EOCRC patients using qRT-PCR.

**Results:**

Fatty Acid Binding Protein 4 (FABP4) was identified as an independent prognostic factor for poor overall survival in EOCRC patients. A prognostic model based on FABP4 demonstrated good predictive accuracy for 1-, 3-, and 5-year survival in both training and validation sets. The high-FABP4 expression was negative correlated TIDE scores, suggesting EOCRC patients with high expression of FABP4 maybe benefit from immunotherapy. Furthermore, 66 chemotherapeutic agents showed significant negative correlations with FABP4 expression. Validation in patient samples confirmed the coordinated upregulation of FABP4 and its associated genes ADIPOQ and IGF1.

**Conclusion:**

FABP4 is a promising independent prognostic biomarker for EOCRC, associated with an immunosuppressive microenvironment and maybe a potential guidance for immunotherapy and chemotherapy selection. Further multi-center prospective studies are warranted to validate its clinical utility.

## Introduction

Colorectal cancer (CRC) is the second leading cause of cancer-related deaths. According to the global cancer statistics in 2022, it is estimated that there will be over 1.9 million new cases of colorectal cancer and 904,000 deaths from colorectal cancer, accounting for approximately one-tenth of all cancer cases and deaths (1). Since the 1950s, the incidence of colorectal cancer has been on the rise (2). Most patients with CRC are diagnosed at an advanced stage, at which point the treatment options are limited and are affected by recurrence, metastasis and drug resistance, resulting in unsatisfactory treatment outcomes (3, 4). The five-year survival rate for advanced colorectal cancer patients worldwide is only about 5% to 10%, and poor prognosis is quite common (5, 6). Many risk factors, such as alcohol consumption, smoking and age, can contribute to the occurrence of CRC (7–9). Early-onset colorectal cancer (EOCRC), also known as young-onset colorectal cancer, refers to cases of colorectal cancer diagnosed in individuals no more that the age of 50 (10). Globally, in many countries, the incidence of age-adjusted EOCRC is increasing at an astonishing rate of 2% to 4% per year (11). Compared with late-onset colorectal cancer (LOCRC), EOCRC exhibits greater invasiveness, higher malignancy, and a more severe clinical stage at the time of diagnosis (12–14). Therefore, there is a critical need to evaluate EOCRC and LOCRC as distinct clinical entities.

Although there have been significant improvements in the diagnosis and treatment of colorectal cancer, the prognosis remains poor, especially for patients in the advanced stage of the disease (1). The immune system plays a crucial role in cancer development, including CRC (15–17). Immune-mediated inflammatory responses can promote tumorigenesis, while cancers develop mechanisms to evade immune surveillance (18, 19). Cancer immunotherapy represents a promising therapeutic avenue that may overcome limitations of conventional chemotherapy and radiotherapy (20, 21). The tumor microenvironment (TME) and immune cells are closely related to the prognosis of patients (22, 23). The tumor microenvironment of CRC is a complex ecosystem composed of various cell types, including tumor cells, fibroblasts, endothelial cells, and immune cells (24). Immune checkpoint inhibitors have been regarded as a breakthrough strategy in cancer immunotherapy. Immune regulatory genes play a crucial role in shaping the TME and influencing treatment responses. For instance, PDL1, a key mediator of immune escape, is expressed on various immune cells, including CD4^+^ and CD8^+^ T cells (25, 26). Immunotherapeutic approaches have shown success across multiple cancer types, including CRC (26–29). Understanding TME, immune cell infiltration, and immune-related gene expression is of great significance in early-onset colorectal cancer.

Age is a risk factor for various cancers (7–9). The incidence of EOCRC is on the rise (11). Immunotherapy has become an extremely promising and effective method in cancer treatment (20, 21). Based on this, the research conducted in this study to investigate immune-related genes as prognostic markers for a unique subgroup of CRC, EOCRC, is of great significance.

## Material and methods

The flow chart was shown in **Supplementary Figure 1**.

### Data acquisition

The gene expression profiles and clinical data for CRC patients were downloaded from the TCGA database and the GEO database. The TCGA, GSE39582, GSE17536, and GSE17537 datasets contained 617, 585, 177, and 55 samples (**Table 1**).

**Table 1:** Clinical features of all CRC.

Data source	Feature		ERCRC(n)	LOCRC(n)
TCGA	Gender	Female	45	244
		Male	37	291
	Stage	Stage1 + 2	31	300
		Stage3 + 4	49	218
GSE39582	Gender	Female	40	223
		Male	38	284
	Stage	Stage1 + 2	40	269
		Stage3 + 4	38	232
GSE17536	Gender	Female	6	75
		Male	15	81
	Stage	Stage1 + 2	7	74
		Stage3 + 4	14	82
GSE17537	Gender	Female	4	25
		Male	5	21
	Stage	Stage1 + 2	0	19
		Stage3 + 4	9	27

The original count data from TCGA were set as the training set. These data were standardized using DEseq2 (R 4.5.1), and then the differential expression of genes was analyzed using limma (R 4.5.1). The data from the same sequencing platform, namely GSE39582, GSE17536 and GSE17537, were set as the validation set, and then the limma (version R 4.5.1) was used for the analysis of gene differential expression. The criterion for screening differentially expressed genes is |Log_2_FoldChange| ≥ 0.3 and P < 0.05.

The clinical samples of 3 EOCRC patients used for the preliminary validation were collected from the gastrointestinal surgery department of Huaihua Central Hospital (formerly Huaihua Second People’s Hospital) from March 2022 to August 2024. All participants signed written informed consent forms, and the collection and use of the clinical samples were approved by the Ethics Committee of Hunan Medical University.

### Cox regression analysis

Univariate and multivariate Cox regression analyses were conducted to identify signatures independently associated with overall survival (OS). The patients with EOCRC were divided into high-risk and low-risk groups based on the Yonden index. Kaplan-Meier (K-M) is used to observe the survival status of the target molecules.

### Evaluation of immune status and immunotherapy

Using the estimate package and ConsensusTME package of R 4.5.1, we conducted an immune status analysis on the data of the training set and the validation set.

The Tumor Immune Dysfunction and Exclusion (TIDE) database was used for analyzing the sensitivity of immunotherapy. TIDE is obtained through the Dysfunction and Exclusion scoring. The differentially expressed genes were normalized according to the requirements of the TIDE database and then used for the analysis of immunotherapy sensitivity.

### Drug response analysis

The differentially expressed genes were filtered by DESeq2. Drug responses in high- and low-risk patient groups were predicted using the “oncoPredict” R package (R4.5.1).

### Pathway enrichment analysis

The Database for Annotation, Visualization, and Integrated Discovery was used for the Kyoto Encyclopedia of Genes and Genomes (KEGG) pathways enrichment analysis based on the differentially expressed genes. Signaling pathway-related gene sets were retrieved from the Molecular Signatures Database (MSigDB), and enrichment scores for each pathway were calculated using the GSEABase, GSVA, dplyr, and tibble packages.

### Validation studies using public data tools

GEPIA 2 (http://gepia2.cancer-pku.cn/#index) was used to identify genes with similar expression patterns across normal colon, colon cancer, normal rectum, and rectal cancer samples. Protein-protein interaction (PPI) networks and functional associations were explored via GeneMANIA (http://genemania.org) and STRING (https://string-db.org), and the resulting interaction networks were visualized using Cytoscape version 3.7.2.

### Quantitative real-time PCR

Total RNA was extracted from tissue samples using the RNA Easy Fast Kit (TIANGEN, China) following the manufacturer’s instructions. Subsequently, 2 μg of total RNA was reverse-transcribed into complementary DNA (cDNA) using the Thermo Scientific RevertAid™ First Strand cDNA Synthesis Kit (Thermo Scientific, USA). Quantitative real-time polymerase chain reaction (qRT-PCR) was performed on a LightCycler^®^ 480 Instrument II (Roche, Switzerland). The sequences of all primers used for qRT-PCR are listed below (**Table 2**).

**Table 2:** The sequences of candidate genes.

Genes		Sequence
FABP4	Forward	ACTGGGCCAGGAATTTGACG
	Reverse	CTCGTGGAAGTGACGCCTT
ADIPOQ	Forward	GGCTGTTGTCATACTTCTCATGG
	Reverse	AACATGCCCATTCGCTTTACC
IGF1	Forward	GCTCTTCAGTTCGTGTGTGGA
	Reverse	GCCTCCTTAGATCACAGCTCC
GAPDH	Forward	GGAGCGAGATCCCTCCAAAAT
	Reverse	GGCTGTTGTCATACTTCTCATGG

### Statistical analysis

Repeated-measures analysis of variance (ANOVA) followed by Bonferroni *post hoc* tests or unpaired two-tailed Student’s t-test was used as appropriate. All statistical data are presented as mean ± standard error of the mean (SEM).

## Results

### Identification of FABP4 as an independent prognostic factor in CRC via differential expression and survival analyses

**Figure 1:**
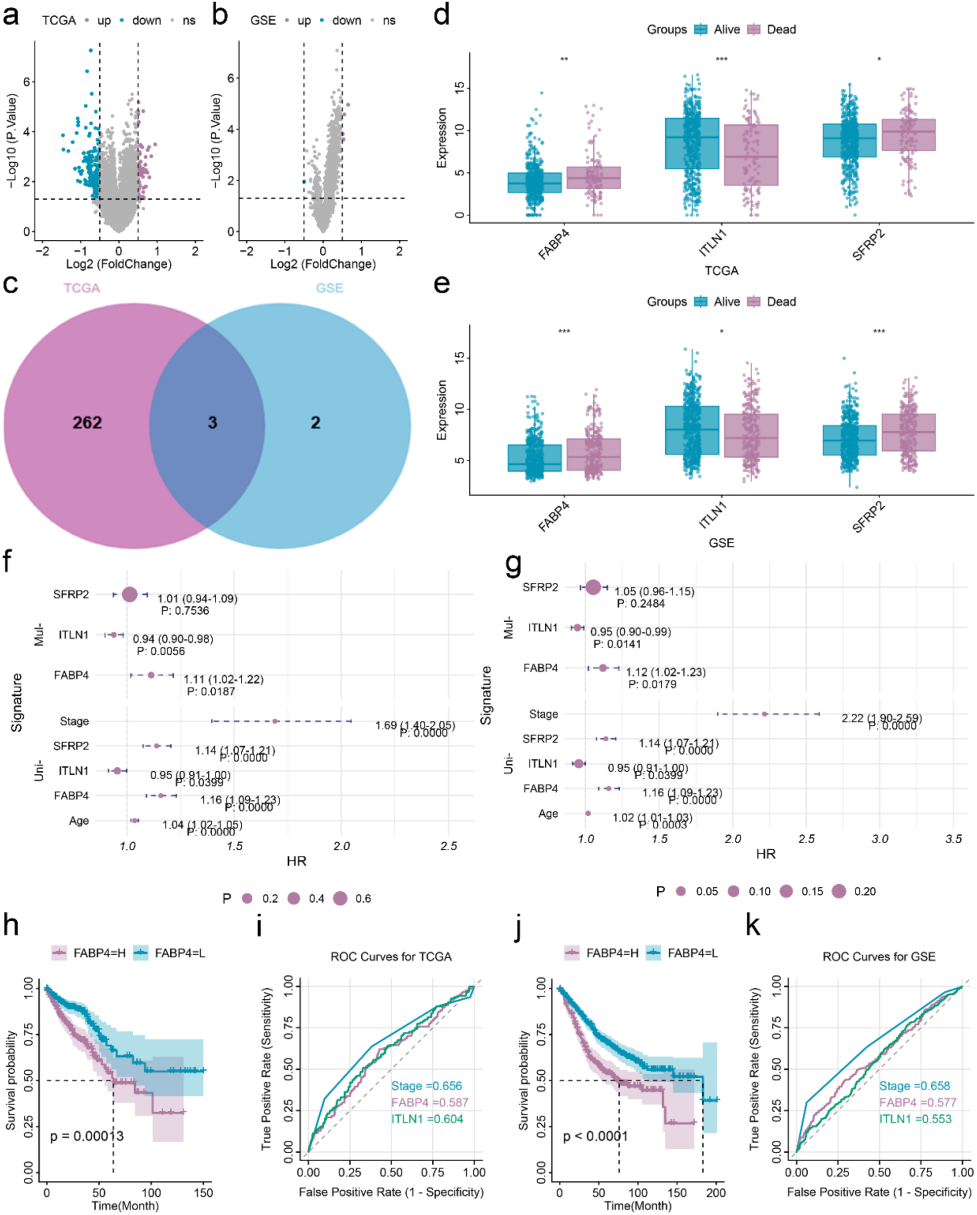
Comprehensive analysis of FABP4 as a prognostic marker for CRC. **(a, b)** volcano plot of differentially expressed genes in CRC patients that survived and those that died in the training set **(a)** and validation set **(b)**. **(c)** Venn diagram illustrating overlapping DEGs across the training set and validation set. **(d, e)**, Expression of three overlap DEGs in the training set **(d)** and validation set **(e)**. **(f, g)** Forest plot of the association between the OS and different clinical features in training **(f)** set and validation set **(g)**. **(h)** K-M curve of FABP4 in training set. **(i)** ROC curve of different clinical features in training set. **(j)** K-M curve of FABP4 in validation set. **(k)** ROC curve of different clinical features in validation set. *p < 0.05, **p < 0.01, ***p < 0.001; NS, not significant.

Differential expression analysis was first performed in CRC patients. A total of 54 upregulated and 4 downregulated differentially expressed genes (DEGs) were identified in the training group, while 211 upregulated and 1 downregulated DEG were found in the validation group (**Figures 1a, b**). Only three DEGs exhibited a consistent expression trend across both groups (**Figure 1c**). In deceased CRC patients, the expression levels of FABP4 and SFRP2 were significantly elevated, whereas ITLN1 expression was markedly reduced (**Figures 1d, e**). Univariate Cox regression analysis indicated that age and disease stage were significantly associated with the overall survival (OS) of CRC. Further univariate and multivariate Cox regression analyses of FABP4, ITLN1, and SFRP2 demonstrated that FABP4 was independently associated with the OS in CRC patients (**Figures 1f, g**). Patients with high FABP4 expression exhibited poorer OS (**Figures 1h, j**). However, the prognostic performance of FABP4 as evaluated by the receiver operating characteristic (ROC) curve was not highly satisfactory (**Figures 1i, k**).

### Construction and validation of FABP4-based prognostic risk model for early-onset CRC

Previous studies have identified age as a risk factor for several cancers (7–9). EOCRC is known to be more aggressive, higher malignancy, and more advanced clinical stages (12–14). In the present study, univariate Cox regression confirmed that age was significantly associated with OS in CRC (**Figures 1f, g**). Chi-square analysis further indicated that EOCRC patients had more advanced disease stages (**Table 3**).

**Table 3:** Analysis of cancer stages in patients with EOCRC and LOCRC.

Data source	Stage	ERCRC(n)	LOCRC(n)	χ²	P
Training	Stage1 + 2	31	300	10.299	0.001
	Stage3 + 4	49	218		
Validation	Stage1 + 2	47	362	2.382	0.123
	Stage3 + 4	61	341		
All	Stage1 + 2	78	662	10.585	0.001
	Stage3 + 4	110	559		

**Figure 2:**
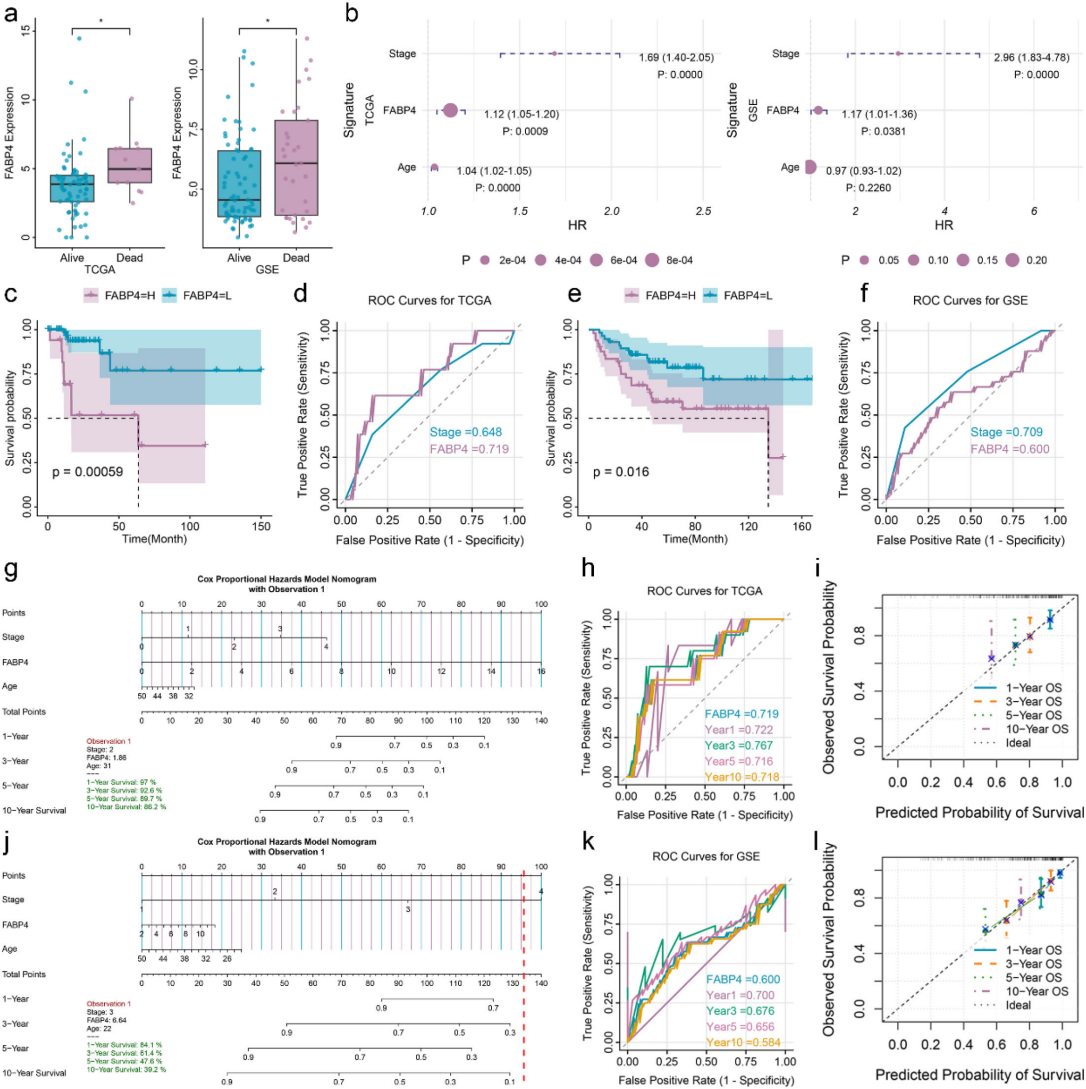
Construction and validation of the FABP4-related prognosis model in EOCRC. **(a)** Expression of FABP4 in the training set (left) and validation set (right). **(b)** Forest plot of the association between the OS and different clinical features in training (left) set and validation set (right). **(c)** K-M curve of FABP4 in training set. **(d)** ROC curve of different clinical features in training set. **(e)** K-M curve of FABP4 in validation set. **(f)** ROC curve of different clinical features in validation set. **(g)** Nomogram diagram of FABP4 model in training set. **(h)** Time-dependent ROC curve of FABP4 in training set. **(i)** The time-dependent calibration curve of FABP4 in the training set. **(j)**, Nomogram diagram of FABP4 model in training set. **(k)** Time-dependent ROC curve of FABP4 in training set. **(l)** The time-dependent calibration curve of FABP4 in the training set. *p < 0.05, **p < 0.01, ***p < 0.001; NS, not significant.

To assess the potential of FABP4 as a biomarker for EOCRC, we first analyzed its expression in surviving and deceased EOCRC patients (**Figure 2a**). FABP4 was significantly elevated in deceased EOCRC patients. Both univariate and multivariate Cox regression analyses confirmed that FABP4 was independently associated with EOCRC prognosis (**Figure 2b**). In the training group, EOCRC patients with high FABP4 expression had significantly worse survival outcomes (**Figure 2c**). The ROC curve analysis yielded an AUC value of 0.719 (**Figure 2d**). Consistent results were observed in the validation set (**Figures 2e, f**). Time-dependent ROC analysis indicated that FABP4 had good predictive efficacy for 1-year survival, with AUCs of 0.722 in the training set and 0.700 in the validation set (**Figures 2g, h, j, k**). Calibration curves revealed a high degree of consistency between predicted and observed outcomes (**Figures 2i, l**).

### FABP4-associated differential gene expression and pathway enrichment analysis in high- vs. low-risk EOCRC

**Figure 3:**
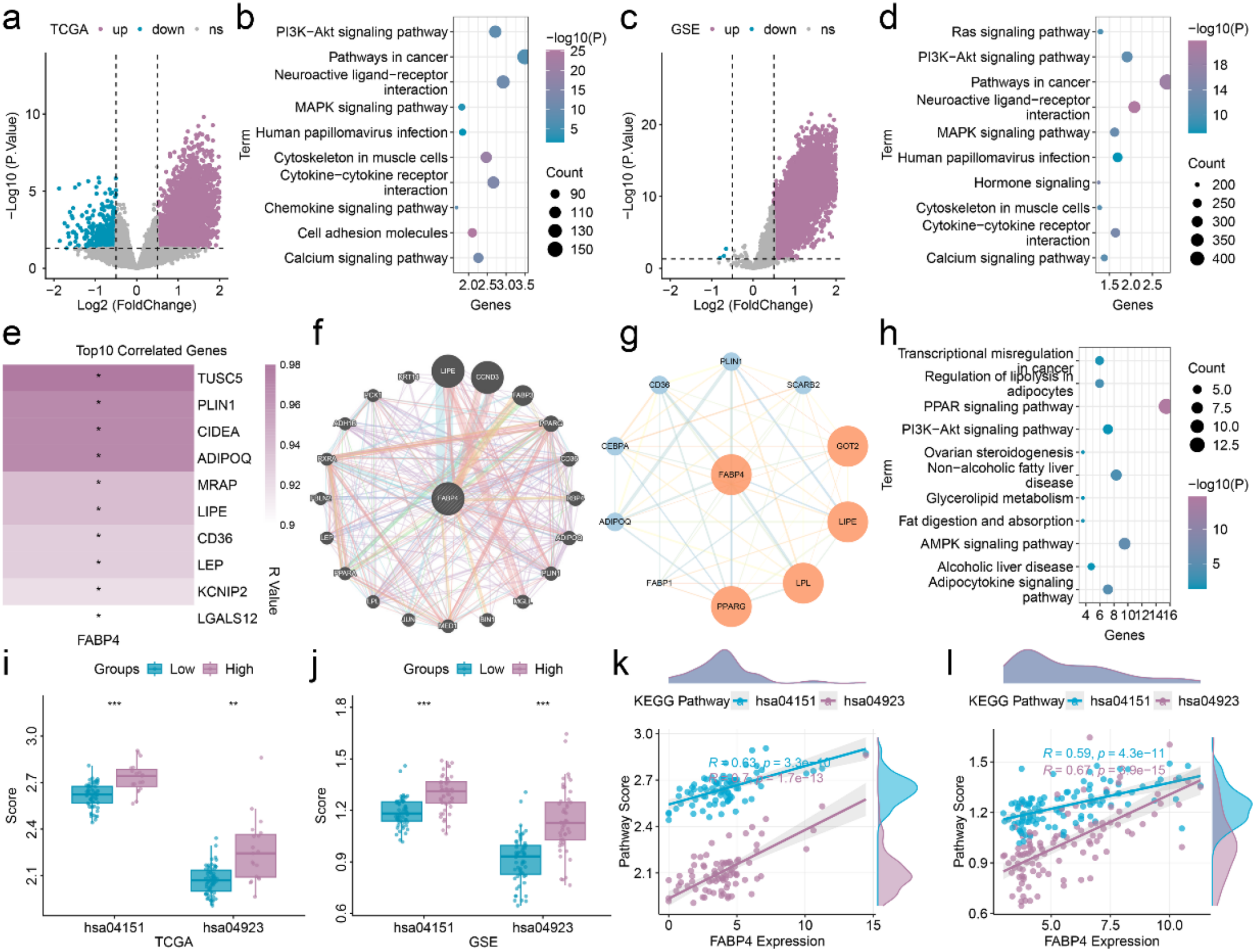
Functional enrichment analysis of EOCRC based on FABP4. **(a)** Volcano plot of DEGs between high- and low-risk groups in the training set. **(b)** Top 10 significantly enriched pathways in the training set. **(c)** Volcano plot of DEGs between high- and low-risk groups in the validation cohort. **(d)** Top 10 significantly enriched pathways in the validation cohort. **(e)** Correlation heatmap of top 10 FABP4 correlated gene filtered by GEPIA2. **(f)** The interaction map of the FABP4 gene was obtained through GENEMANIA screening. **(g)** The interaction map of the FABP4 gene was obtained through STRINIG screening. **(h)** The 11 signaling pathway genes obtained through significant enrichment of FABP4-related genes. **(i, j)** different analysis of different signaling pathway between low- and high- EOCRC patients in training set **(i)** and validation set **(j)**. **(k, l)** Correlation of FABP4 with the different signaling pathway in training set **(k)** and validation set **(l)**. *p < 0.05, **p < 0.01, ***p < 0.001; NS, not significant.

FABP4 may serve as an independent prognostic biomarker for EOCRC. To elucidate the role of FABP4 in EOCRC progression, we performed gene expression analysis based on FABP4 expression levels. In the training set, high-risk EOCRC patients exhibited significantly elevated expression of 3,799 genes and decreased expression of 552 genes (**Figure 3a**). Enrichment analysis of those DEGs identified 88 significantly enriched signaling pathways, with the top 10 shown in **Figure 3b**. In the validation set, high-risk EOCRC patients showed significantly increased expression of 19,019 genes and decreased expression of 3 genes (**Figure 3c**). Pathway enrichment analysis of those DEGs revealed 157 significantly enriched signaling pathways, with the top 10 presented in **Figure 3d**.

Additionally, we performed correlation analyses using publicly available databases. Analysis based on the GEPIA2 database identified the top 100 correlated genes. Top 10 correlated genes were showed in **Figure 3e**. The GENEMANIA database revealed 20 FABP4-related genes (**Figure 3f**). Protein-protein interaction analysis using the STRING database identified 10 FABP4-associated proteins (**Figure 3g**). Integration of results from these three databases yielded 120 FABP4-related genes. KEGG pathway enrichment analysis identified 11 significantly enriched signaling pathways (**Figure 3h**). Among these pathways, the PI3K-Akt signaling pathway and regulation of lipolysis in adipocytes were consistently enriched across all three datasets. The pathway scores for these two signaling pathways were significantly higher in high-risk EOCRC patients (**Figures 3i, j**) and showed significant correlation with FABP4 expression (**Figures 3k, l**).

### Immunotherapeutic and chemotherapeutic prediction of the FABP4-associated risk model in EOCRC

**Figure 4:**
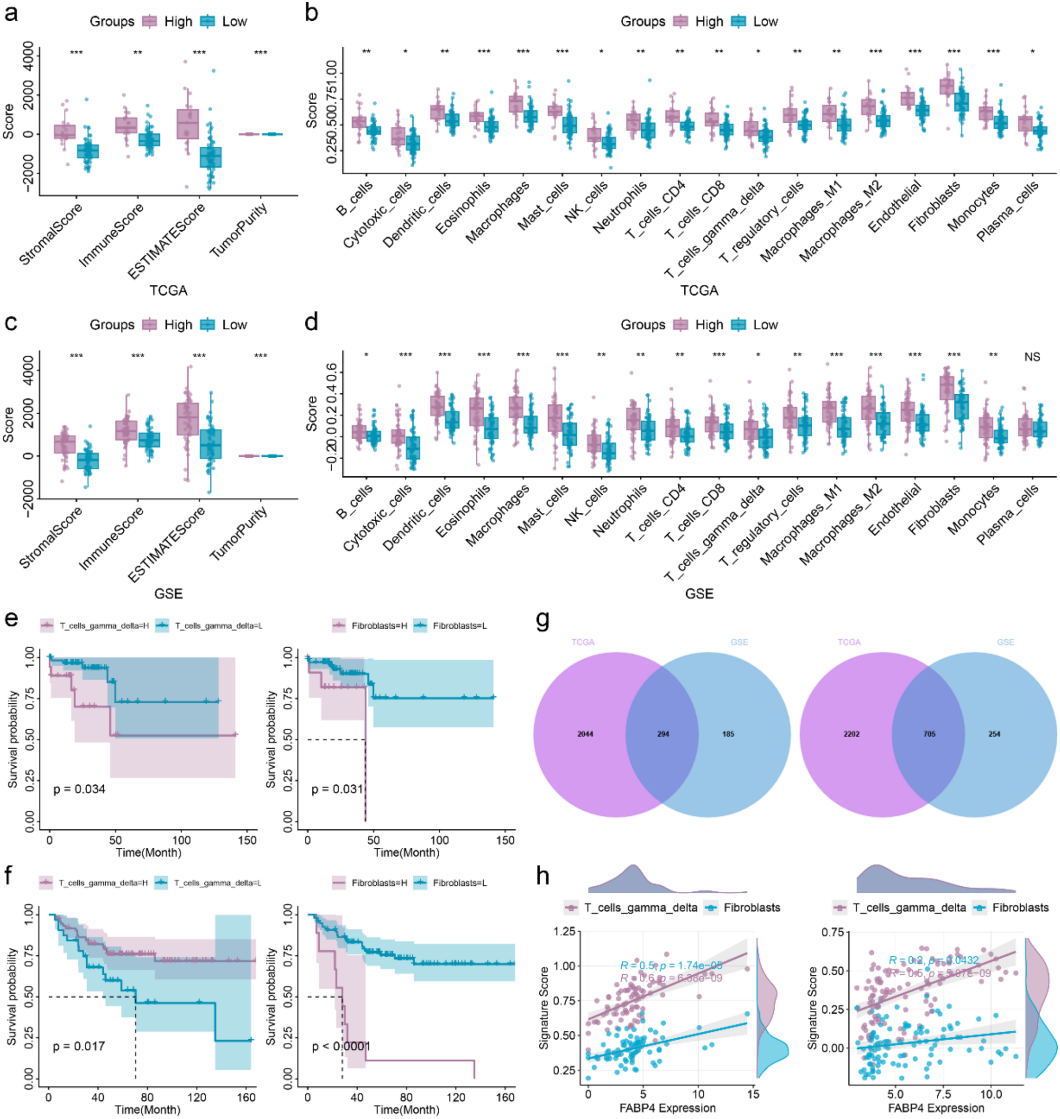
Immune landscape of EOCRC patients stratified by risk score. **(a, b)** Comparison of immune microenvironment **(a)** and different immune cells **(b)** between high- and low-risk EOCRC patients in the training set. **(c, d)** Comparison of immune microenvironment **(c)** and different immune cells **(d)** between high- and low-risk EOCRC patients in the validation set. **(e)** K-M curve of T_cells_gamma_delta in the training set (left) and validation set (right). **(f)** K-M curve of Fibroblasts in training set (left) and validation set (right). **(g)** Overlap genes related to T_cells_gamma_delta (left) and Fibroblasts (right) obtained through Venn diagram analysis. **(h)** The linear correlation of T_cells_gamma_delta (left figure) and fibroblasts (right figure) with FABP4. *p < 0.05, **p < 0.01, ***p < 0.001; NS, not significant.

Given that FABP4 is an immune-related gene, we investigated whether its aberrant expression is associated with the immune status of EOCRC. We therefore performed differential analysis of the immune microenvironment in EOCRC patients. In the training set, high-risk EOCRC patients exhibited significantly increased stroma, immune, and ESTIMATE scores, along with significantly decreased tumor purity (**Figure 4a**). The infiltration scores of most immune cell types were also significantly elevated (**Figure 4b**). Consistent results were observed in the validation set for both immune microenvironment signatures scores and immune cell infiltration score (**Figures 4c, d**). K-M curve analysis showed the infiltration of T_cells_gamma_delta and Fibroblasts were significantly correlated with the OS of EOCRC (**Figures 4e, f**). Correlation analysis showed 294 differentially expressed genes (DEGs) were identified to be associated with the immune infiltration of T_cells_gamma_delta (**Figure 4g**). 705 differentially expressed genes (DEGs) were identified to be significantly correlated with the immune infiltration of Fibroblasts. The correlation of T_cells_gamma_delta and Fibroblasts with FABP4 were showed in **Figure 4h**.

**Figure 5:**
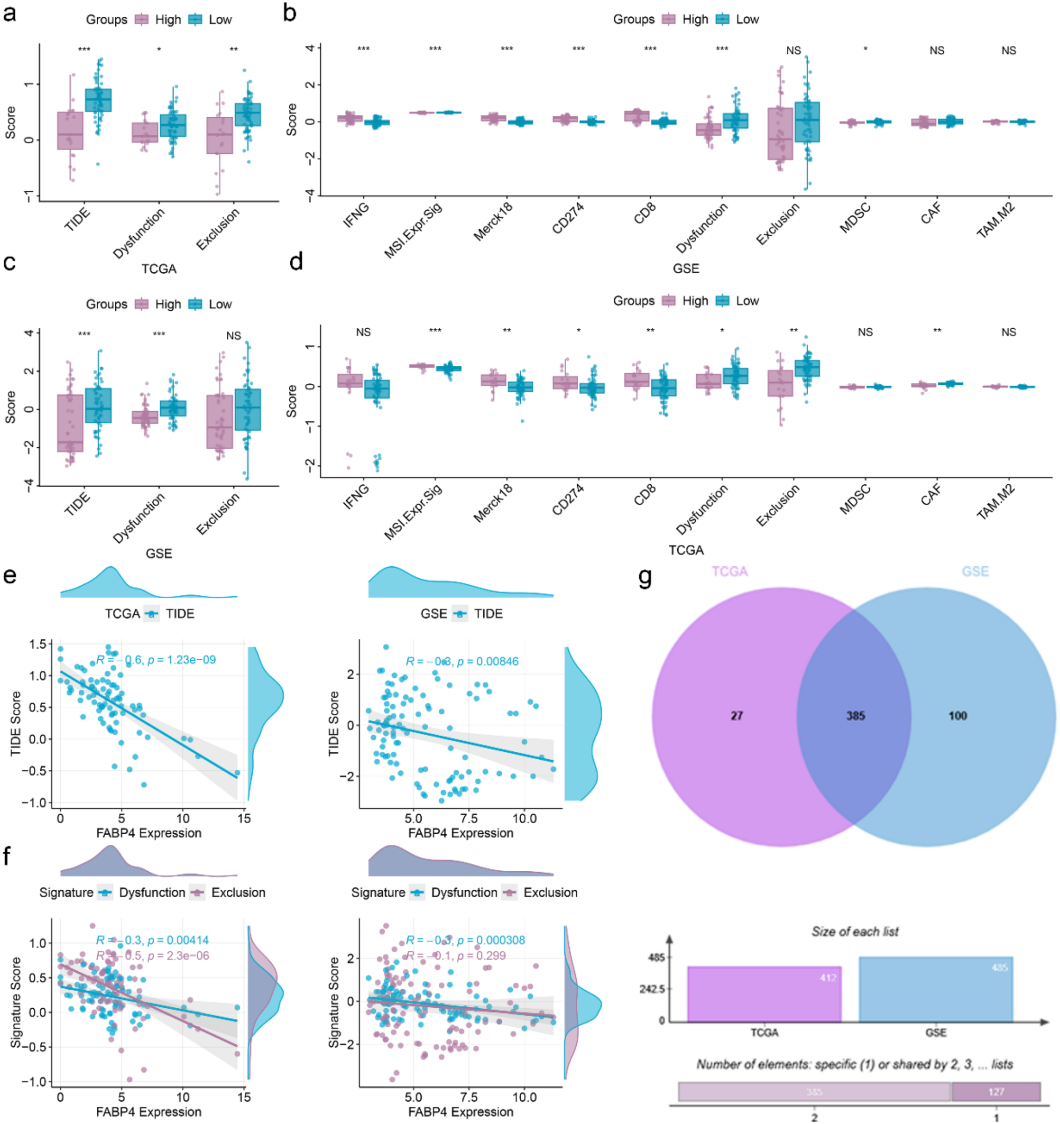
Analysis of immunotherapy sensitivity based on FABP4. **(a, b)** Analysis of immunotherapy related signature **(a)** and molecules **(b)** in training set. **(c, d)** Analysis of immunotherapy related signature **(c)** and molecules **(d)** in validation set. **(e)** The linear correlation of FABP4 with TIDE in the training set (left) and validation set (right). **(f)** The linear correlation of dysfunction and exclusion with TIDE in the training set (left) and validation set (right). **(g)** Overlap genes related to TIDE obtained through Venn diagram analysis. *p < 0.05, **p < 0.01, ***p < 0.001; NS, not significant.

To assess whether FABP4 could guide immunotherapy selection, we performed TIDE (Tumor Immune Dysfunction and Exclusion) analysis based on DEGs between high- and low-risk EOCRC patients. EOCRC patients showing immune response exhibited significantly increased TIDE scores (**Supplementary Figures 2a, c**). Multiple immunotherapy-related molecules showed significant differences between responders and non-responders (**Supplementary Figures 2b, d**). While high-risk EOCRC patients demonstrated significantly decreased TIDE scores (**Figures 5a, c**). Multiple immunotherapy-related molecules showed significant differences between high- and low-risk EOCRC patients (**Figures 5b, d**). FABP4 expression was significantly negatively correlated with TIDE scores (**Figure 5e**). Further analysis revealed that FABP4 was significantly negatively correlated with the “dysfunction” component of TIDE, but not significantly correlated with “exclusion” (**Figure 5f**). Correlation analysis showed 385 DEGs were determined to be correlated with the TIDE score (**Figure 5g**).

Additionally, to evaluate the potential of FABP4 in personalizing EOCRC treatment strategies, we compared the sensitivity of various chemotherapeutic drugs between risk groups. The training set identified 108 drugs with significantly different sensitivities between high- and low-risk patients, while the validation set identified 141 such drugs. Across both datasets, 104 drugs showed consistent sensitivity differences. Correlation analysis indicated that 66 drugs were significantly negatively correlated with FABP4 expression, suggesting that EOCRC patients with high FABP4 expression may benefit from these 66 agents. Drug sensitivity profiles and their correlations are presented in **Supplementary Figures 3a–d**.

### Clinical validation of FABP4 and Its related genes

**Figure 6:**
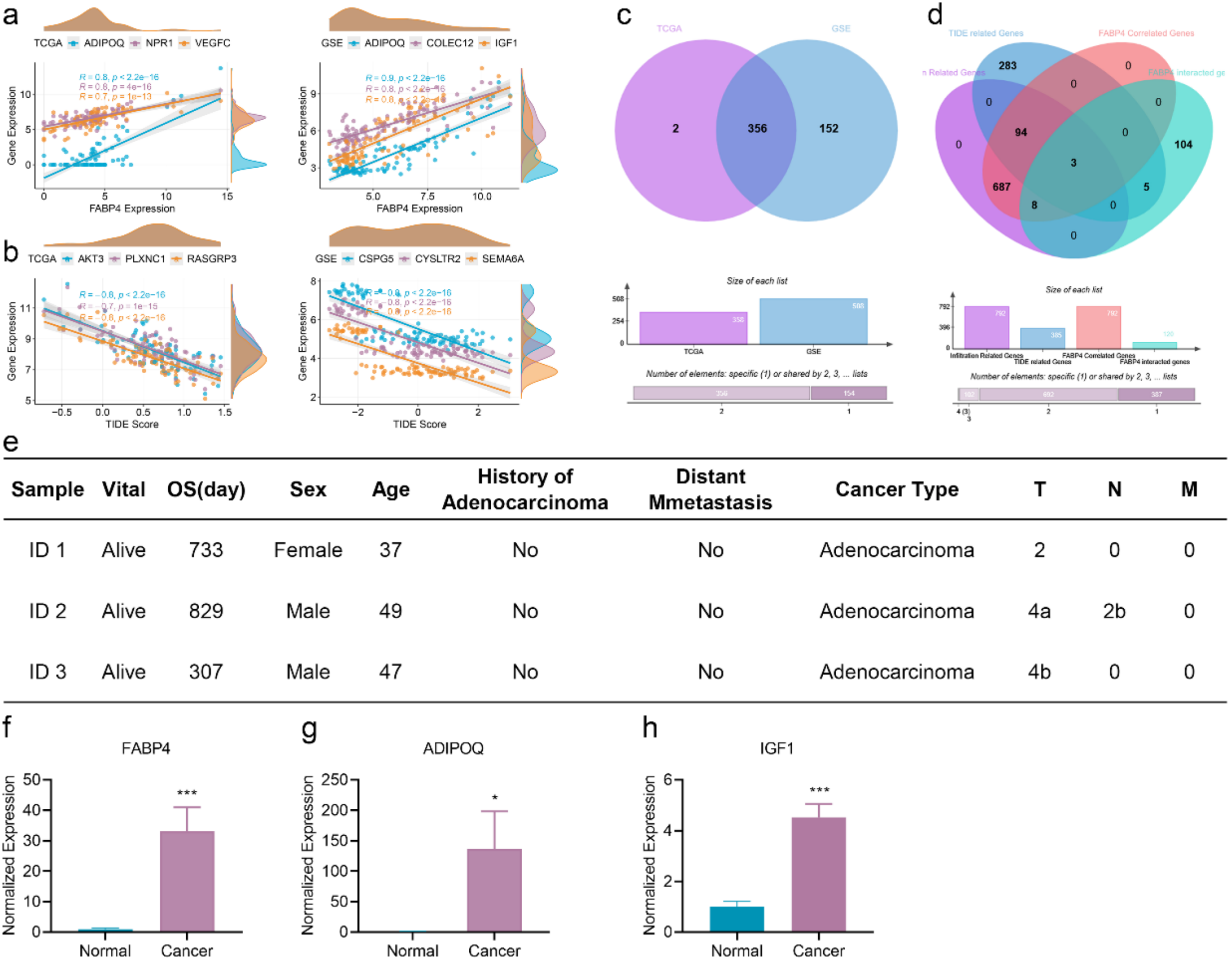
Validation of FABP4 and its related genes. **(a)** The top 3 correlated IRDEGs of FABP4 in the training set (left) and validation set (right). **(b)** The top 3 correlated IRDEGs of TIDE in the training set (left) and validation set (right). **(c)** Overlap genes related to FABP4 obtained through Venn diagram analysis. **(d)** A Venn diagram of four gene sets revealed three common immune-related differentially expressed genes (IRDEGs). The gene sets included those for immune infiltrating cells, TIDE, and FABP4 (from both the present study and a network analysis). **(e)**, Clinical information of EOCRC. **(f–h)** T-test analysis of differential expression of FABP4 **(f)** ADIPOQ **(g)** and IGF1 **(h)** in normal tissues and ovarian cancer metastatic tissue. N = 3. *p < 0.05, **p < 0.01, ***p < 0.001; NS, not significant.

In the training and validation sets, there were 4,351 and 19,022 DEGs between high- and low-risk EOCRC patients, respectively (**Figures 4a–c**). Among these, 522 immune-related genes exhibited consistent expression patterns. The three most significantly correlated IRDEGs are shown in **Figures 6a, b**. Correlation analysis identified 356 IRDEGs were determined to be correlated with FABP4 (**Figure 6c**). The Venn diagram analysis of the four datasets immune infiltrating cell-related genes, TIDE-related genes, FABP4-related genes (derived from the present analysis) and FABP4-related genes (derived from network analysis) revealed three overlapping IRDEGs (**Figure 6d**). To experimentally validate these findings, surgical specimens were collected from three EOCRC patients. Clinical characteristics of these patients are summarized in **Figure 6e**. Expression analysis confirmed significant upregulation of FABP4 in cancer tissues from EOCRC patients (**Figure 6f**). Similarly, the other two FABP4-associated IRDEGs, ADIPOQ and IGF1, also showed significantly elevated expression in EOCRC cancer tissue (**Figures 6g, h**).

## Discussions

CRC is one of the most common malignant tumors worldwide and is the leading cause of cancer-related morbidity and mortality. Multiple risk factors contribute to the formation of colorectal cancer, including older age, a family history of colorectal cancer, personal medical history (such as inflammatory bowel disease), and modifiable lifestyle factors, etc (30). In recent years, many cancers have shown a trend of younger onset of disease, including colorectal cancer. Moreover, it has been found that these early-onset cancers have more aggressive nature, higher malignancy, and more severe clinical stages. Therefore, screening for prognostic biomarkers of EOCRC is of great practical significance for its diagnosis and treatment.

In order to identify the appropriate biomarkers for EOCRC, we obtained 82 cases of EOCRC from the TCGA database as the training set, and 106 cases of EOCRC from the GSE39582, GSE17536, and GSE17537 databases as the validation set. After a series of comprehensive analyses, FABP4 was identified as a prognostic marker for EOCRC. It was also found that FABP4 has a significant negative correlation with the sensitivity to immunotherapy and a negative correlation with 66 chemotherapy drugs, suggesting that they may serve as treatment options for EOCRC patients with high FABP4 expression.

Fatty acid binding proteins (FABPs) are a family of small cytoplasmic proteins that specialize in binding and transporting hydrophobic fatty acids. As a member of this family, FABP4 is highly expressed in adipose tissue and macrophages (31). It plays critical roles in the binding, metabolism, and transport of long-chain fatty acids, particularly in the contexts of inflammation, atherosclerosis, and metabolic syndrome (32–34). Previous studies have reported elevated FABP4 expression in metastatic cancer cells (32), with a notable enrichment at the interface between primary ovarian cancer cells and adipocytes (35). Upregulation of FABP4 has also been observed in prostate cancer and breast cancer (36–38). In CRC specifically, Zhang et al. reported that preoperative patients had higher serum FABP4 levels compared to healthy controls, and these levels decreased significantly two weeks after surgical resection (39). Zhang et al. also found that the average concentration of plasma FABP4 in patients with colorectal cancer was higher than that in the control group. The concentration of plasma FABP4 is related to the tumor, lymph node, and metastasis (TNM) stage as well as lymph node metastasis (40). Pan et al. found suppress the expression of FABP4 could inhibited the migration and invasion ability of CRC (41). Kim et al. found that the expression of FABP4 was significantly correlated with the reduction in overall survival rate and recurrence-free survival rate. The expression of FABP4 was also significantly associated with the aggressive behavior and pathological characteristics of the tumor (42). In this study, we found that FABP4 was significantly elevated in EOCRC and was significantly correlated with PI3K-Akt. These results not only further confirm the role of FABP4 in CRC, but more importantly, FABP4 also plays a significant role in EOCRC, which has more aggressive nature, higher malignancy, and more severe clinical stages. Our research has further expanded the significance of FABP4.

FABP4 demonstrated ideal performance in predicting the prognosis of patients with EOCRC over a period of 1 year, 3 years, and 5 years. In the training set, the AUC values were 0.719, 0.722, and 0.767, respectively. In the validation set, the AUC values were 0.700, 0.676, and 0.656. The calibration curve also shows that FABP4 has relatively consistent characteristics. Li et al. constructed the Nomogram model for EOCRC and obtained the AUC values for 3 years, 5 years and 10 years in the training group: 78.74%, 76.21%, 73.19%; in the validation group: 78.99%, 77.28%, 72.97%; and in the external validation group: 73.49%, 77.90%, 73.60% (43). Xiang et al. constructed a prognostic model for EOCRC using MCM2, INHBA, CGREF1 and KLF9. The area under the curve values for 1-year, 3-year and 5-year survival rates in the training set and validation set were 0.687/0.663, 0.764/0.798, and 0.730/0.792, respectively (44). In this study, we found that FABP4 is a prognostic biomarker for EOCRC. When FABP4 was used solely as a prognostic indicator for ovarian and colorectal cancers, we found that its prognostic accuracy was comparable to that of other models. This is also one of our advantages - that we only use one prognostic marker. Therefore, in subsequent studies, we can consider integrating FABP4 with other candidate markers and models to achieve higher accuracy.

The immune system plays a crucial role in the development of cancer. Previously, a large number of studies on immune-related prognostic markers for CRC have been conducted (15–17). Many immune-related markers have been screened and identified (45), such as ESM1, SPP1, etc (46, 47). As an immune-related gene, FABP4 may also provide insights to guide immunotherapy strategies. Analysis of three surgically resected EOCRC tissue samples confirmed the upregulated expression of FABP4 and two IRDEGs, ADIPOQ, IGF1. IGF1 has been implicated in the pathogenesis of numerous cancers and is considered a promising therapeutic target (48, 49). In the research on the immune microenvironment, Pan et al. found that by conducting immunohistochemical examinations on tissue microarray samples from 35 CRC patients, they observed the expressions of FABP4 and CD8. This indicated that in patients with elevated FABP4 expression, there was an increase in CD8 infiltration. These findings suggest that FABP4 is closely related to the immune response and metastasis, and may potentially serve as a therapeutic target for CRC (41). In this study, we also discovered that FABP4 is not associated with CD8 cell infiltration, but is almost related to the infiltration of all immune cells. In the corresponding analysis of immunotherapy, the results of Pan et al.’s research suggested that FABP4 might serve as a therapeutic target for CRC (41). Xiao et al. found that high SPP1 expression was associated with more neutrophil infiltration and less CD8+ T cell infiltration (47). In this study, based on the results of bioinformatics analysis, we suggested that FABP4 is significantly negatively correlated with the key indicators of immune therapy, TIDE, from a quantitative perspective, further confirming the relationship between FABP4 and immunotherapy.

Of course, our current research also has some limitations, which need to be acknowledged and further improved. Firstly, the accessibility of the EOCRC samples is relatively limited, which restricts our research subjects to only 208 existing cases and 3 prospectively collected samples. Therefore, our research results need to be verified in larger-scale, multi-center prospective studies to confirm their general applicability. Secondly, more *in vitro* and *in vivo* functional experiments are needed to clarify the exact mechanism of action of FABP4 and its related genes in the progression of EOCRC, and whether FABP4 really affects the efficacy of immunotherapy and other issues still require further research.

## Conclusion

Our present study identifies FABP4 as a novel, independent prognostic biomarker for EOCRC. High FABP4 expression is significantly associated with poorer overall survival and demonstrates good predictive value for patient outcomes. Mechanistically, FABP4 likely promotes EOCRC progression via the PI3K-Akt signaling pathway. Furthermore, it is linked to immunosuppressive tumor microenvironment signatures and TIDE score, suggesting may utility in predicting immunotherapy response. The risk model based on FABP4 also identified 66 chemotherapeutic drugs that are significantly correlated with FABP4, which may be used for personalized treatment of patients with high FABP4 expression. The coordinated upregulation of FABP4 with ADIPOQ, LEP, and IGF1 in patient samples underscores their collective role. While these findings highlight the significant prognostic and therapeutic value of FABP4 in EOCRC, further multi-center prospective studies are warranted for clinical validation.

## Data Availability

The original contributions presented in the study are included in the article/**Supplementary Material**. Further inquiries can be directed to the corresponding authors.
