# Supplementary material for: Unveiling the prognostic role of FABP4 in early-onset colorectal cancer through big data analysis and preliminary clinical validation
Source: Front Oncol. 2026 Jan 13;15:1689952. doi: 10.3389/fonc.2025.1689952 (PMC12836056; doi:10.3389/fonc.2025.1689952)
Supplement: Supplementary file 1 [file DataSheet1.pdf]

Unveiling the Prognostic Role of FABP4 in Early-Onset Colorectal Cancer through Big Data Analysis and Preliminary Clinical Validation

Yu Wu[1#], Weiwei Zou[2#], Shengjun Zhang[2#], Lipeng Zhao[2], Shaohua He[2], Fan Yao[1], Peilin Qing[1], Yixin Li[1], Jie Li[1], Xiao-Liang Xing[1*].

[1] Hunan University of Medicine General Hospital, School of Public Health and Emergency Management, School of Medical Laboratory Science, Hunan University of Medicine, Huaihua 418000, Hunan, P. R. China.

[2] Huaihua Central Hospital, Huaihua 418000, Hunan, P. R. China.

[#]Contributes equally to this work.

*Correspondence author: Xiao-Liang Xing, xiaoliangxing@hnmu.edu.cn.

Supplementary information: three figures.

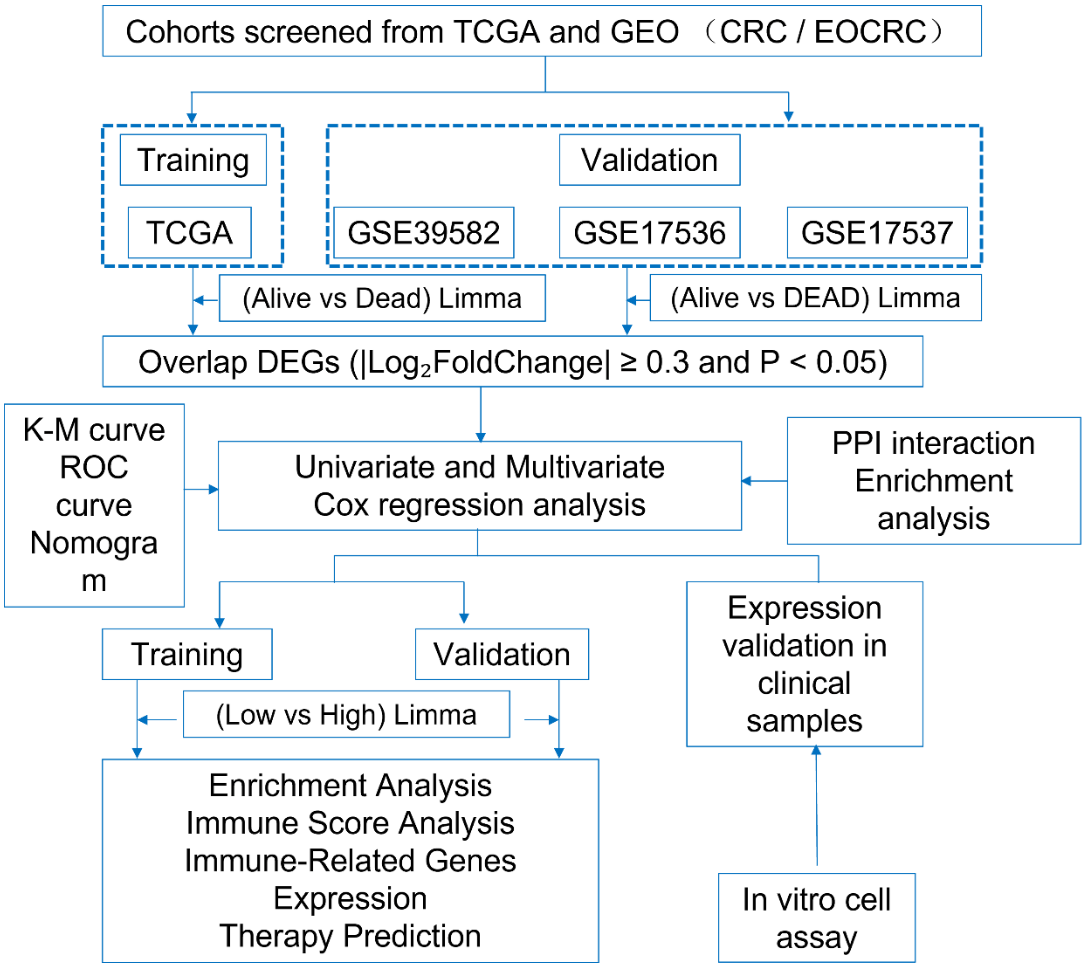

Supplementary Figure 1 Flowchart of the bioinformatics analysis process.

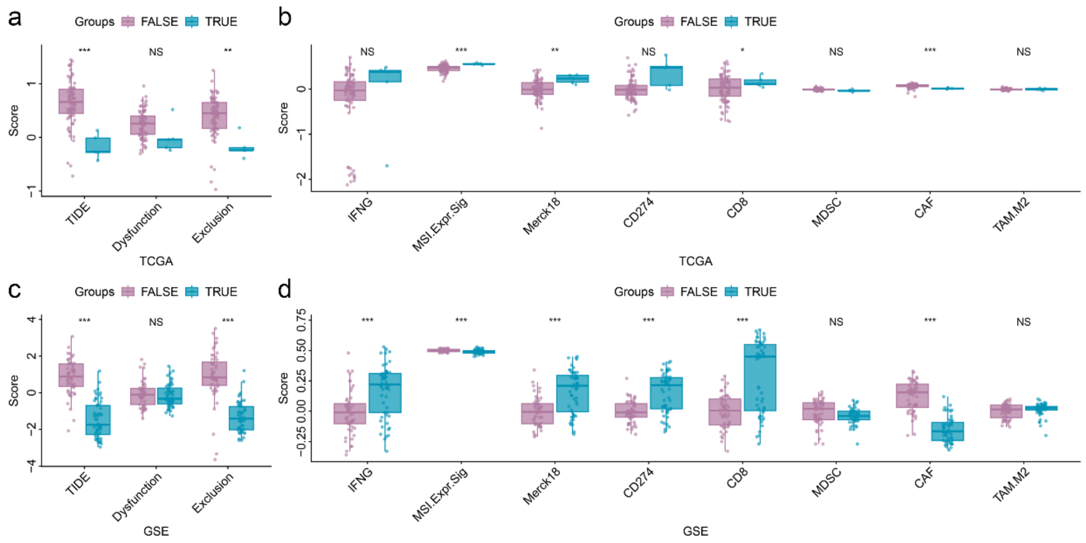

Supplementary Figure 2 Evaluation of the risk model in EOCRC.

a-b, Comparison of immune microenvironment (a) and different immune cells (b) between response- and nonresponse-EOCRC patients in the training set. **c-d,** Comparison of immune microenvironment (c) and different immune cells (d) between response- and nonresponse-EOCRC patients in the validation set.

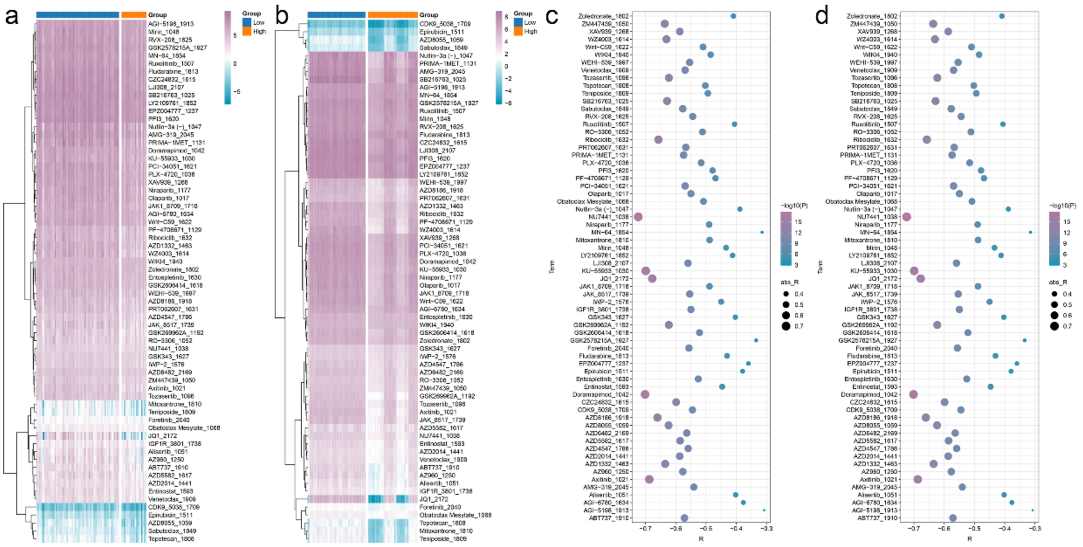

Supplementary Figure 3 Enrichment analysis of FABP4-related Signatures.

a, Heat maps of 66 drug sensitivity in training set. **b,** Heat maps of 66 drug sensitivity in validation set. **c,** Bubble chart showing the correlation between 66 drug sensitivity values and FABP4 in the training set. **c,** Bubble chart showing the correlation between 66 drug sensitivity values and FABP4 in the validation set.
